# Vitamin D Deficiency (VDD) and Susceptibility towards Severe Dengue Fever—A Prospective Cross-Sectional Study of Hospitalized Dengue Fever Patients from Lahore, Pakistan

**DOI:** 10.3390/tropicalmed8010043

**Published:** 2023-01-05

**Authors:** Somia Iqtadar, Amjad Khan, Sami Ullah Mumtaz, Shona Livingstone, Muhammad Nabeel Akbar Chaudhry, Nauman Raza, Mehreen Zahra, Sajid Abaidullah

**Affiliations:** 1 Department of Medicine, King Edward Medical University, Lahore 54000, Pakistan; 2 Nuffield Division of Clinical Laboratory Sciences, Radcliffe Department of Medicine, John Radcliffe Hospital, University of Oxford, Headley Way, Oxford OX3 9DU, UK; 3 School of Medicine, University of Dundee, Ninewells Hospital, Dundee DD1 9SY, UK; 4 Cath Laboratory, Punjab Institute of Cardiology, Lahore 54000, Pakistan

**Keywords:** vitamin D deficiency, dengue fever, dengue hemorrhagic fever, dengue shock syndrome

## Abstract

Dengue is a mosquito-borne flaviviral serious febrile illness, most common in the tropical and subtropical regions including Pakistan. Vitamin D is a strong immunomodulator affecting both the innate and adaptive immune responses and plays a pivotal role in pathogen-defense mechanisms. There has been considerable interest in the possible role of vitamin D in dengue viral (DENV) infection. In the present prospective cross-sectional study, we assessed a possible association between serum vitamin D deficiency (VDD) and susceptibility towards severe dengue fever (DF) illness. Serum vitamin D levels were measured at the time of hospitalization in 97 patients diagnosed with dengue fever (DF), dengue hemorrhagic fever (DHF) or dengue shock syndrome (DSS) at Mayo Hospital, King Edward Medical University, Lahore, PK, from 16 November 2021 to 15 January 2022. In terms of disease severity, 37 (38.1%) patients were DF, 52 (53.6%) were DHF grade 1 and 2, and 8 (8.2%) were DSS. The results revealed that most patients (75 (77.3%)) were vitamin-D-deficient (i.e., serum level < 20 ng/mL), including 27 (73.0%) in DF, 41 (78.8%) in DHF grade 1 and 2, and 7 (87.5%) in DSS. The degree of VDD was somewhat higher in DSS patients as compared to DF and DHF grade 1 and 2 patients. Overall, serum vitamin D levels ranged from 4.2 to 109.7 ng/mL, and the median (IQR) was in the VDD range, i.e., 12.2 (9.1, 17.8) ng/mL. Our results suggest that there may be a possible association between VDD and susceptibility towards severe dengue illness. Hence, maintaining sufficient vitamin D levels in the body either through diet or supplementation may help provide adequate immune protection against severe dengue fever illness. Further research is warranted.

## 1. Introduction

Dengue is a mosquito-borne flaviviral serious febrile illness, most common in the tropical and subtropical regions, especially in Southeast Asia, Latin America, the Caribbean, and the Pacific Islands. Dengue virus (DENV) has four serotypes (DENV1, DENV2, DENV3 and DENV4), and the first infection with one of the four serotypes is usually mild or asymptomatic, while a second infection with one of other serotypes may cause severe dengue manifestations. Each year, an estimated 50 million people are affected by DENV infection in dengue-endemic countries, with 70% of cases reported in Asia [1]. Each year, Pakistan also experiences large dengue outbreak that affect the healthcare system. Although the global fatality rate from dengue is very low, i.e., 1% [2], without timely management it can increase up to 20% [3]. The clinical manifestation of dengue infection ranges from asymptomatic to a wide range of symptoms, known as “dengue fever (DF)”. Symptoms of DF range from a mild flu-like syndrome to a severe form, dengue hemorrhagic fever (DHF). DHF is characterized by hemorrhagic manifestations such as spontaneous bleeding, significant decrease in platelet count and increased vascular permeability noted as increased hemo-concentration or pleural effusion or ascites [4]. DHF, if not timely managed, may progress to the life-threatening stage of hypovolemic shock, known as dengue shock syndrome (DSS) [5]. DSS occurs at the time of or shortly after blood pressure drop, and is characterized by a rapid, weak pulse, narrow pulse pressure (≤20 mm Hg) or hypotension with cold, clammy skin in the early stage of shock. In the absence of a prompt and appropriate treatment, this may soon progress to more serious form of shock in which pulse and blood pressure become undetectable, resulting in death within 12 to 36 h after the onset of shock. 

A dengue virus vaccine named Dengvaxia^®^ (CYD-TDV) [6] has been recently developed and is licensed in 20 countries, but with limited use. In the EU and the United States, Dengvaxia^®^ has been approved for use by individuals living in endemic areas, aged 9–45 years and who had a previous dengue virus infection, i.e., prevention of secondary dengue infection [7,8]. In Pakistan, there is currently no dengue vaccine available to help provide protection against DENV infection. In the absence of a specific antiviral treatment, the present management of dengue fever illness is primarily supportive. Currently, the standard care is limited to rest, and administration of antipyretics such as paracetamol when fever is too high. In addition, crystalloid fluids, for maintaining fluid electrolyte balance and colloids to increase intravascular volume along with blood and blood products in case of bleeding are used in the management of dengue infection. 

The reason why some patients progress to severe and potentially fatal forms of dengue illness remains unknown. Several host-associated risk factors including prior exposure to DENV, co-morbidity, and genetic predisposition are believed to influence the likelihood of developing severe dengue illness [9]. In the absence of DENV specific antiviral therapy, and limited therapeutic arsenal, identifying host’s new potentially modifiable risk factors that can prevent progression to severe illness, is currently under investigation. 

The host’s nutritional status has been suggested as a potentially relevant predictor of disease progression in dengue patients [10] because some nutrients possess strong immunomodulatory activity. There is a growing body of evidence to suggest vitamin D deficiency (VDD), i.e., serum level < 20 ng/mL, as possible risk factor for developing severe dengue illness. Though, limited studies have evaluated the possible association between VDD and dengue disease severity [11,12,13,14,15,16,17], but the available evidence is scanty and inconsistent. Elucidating the possible link between VDD and severity of dengue symptoms would constitute a critical first step to investigate supplementation of this nutrient as a preventive against susceptibility of developing severe dengue illness. In the present study, we evaluated systemic vitamin D, i.e., serum 25-(OH) D levels in adults hospitalized with DF, DHF and DSS [18] prospectively upon admission at our institution, the largest tertiary teaching hospital in Lahore, Pakistan. We aimed to assess a possible association between low systemic 25-(OH) D levels and susceptibility towards severe dengue fever. 

## 2. Materials and Methods

### 2.1. Study Design

This was a prospective, single-center, single-group, cross-sectional study conducted at the Department of Medicine, King Edward Medical University (KEMU), Lahore, PK. The study aimed to assess possible association between VDD and susceptibility toward severe DENV illness, evaluated at the time of admission, in hospitalized patients diagnosed with DF, DHF or DSS. 

### 2.2. Eligibility Criteria 

NS1/IgM dengue positive patients with platelet count < 100,000/µL of blood, of either gender who were admitted to the dengue wards, High-dependency Units (HDUs) and Intensive Care Units (ICUs) of Mayo Hospital, King Edward Medical University, from 16 November 2021 to 15 January 2022 for diagnosis of DENV fever, were included in the study. Participants gave informed written consent to participate in the study. The study was approved by the Institution Review Board, King Edward Medical University, Lahore, PK via Ref. number 351/RC/KEMU. 

### 2.3. Treatment 

Upon hospitalization, acetaminophen was used to relieve fever and body pain, while antiemetics including dompdridone and ondansetron were used to treat nausea and vomiting. Patients were treated with 2.5 L/24 h of oral fluid or intravenous normal saline as maintenance fluid. Severe disease was managed in HDUs and ICUs with judicious fluid administration calculated according to weight of the patient, with a maximum of 4.6 L in entire 48 h of critical phase. Crystalloids were mainstay of treatment to maintain adequate hydration to avoid both shock and fluid overload and ensure organ perfusion. Fluids were administered as oral or intravenous (IV) infusion (depending on the oral tolerance of patient), aiming for a pulse pressure of more than 30 and urine output of 0.5 mL/kg/hour while carefully monitoring vital statistics and hematocrit. In selective cases of DHF or DSS, colloids, blood and blood products were also used.

### 2.4. NS1/IgM and Vitamin D/25-(OH)D Assays

NS1/IgM dengue detection was carried out by enzyme-linked immunosorbent assay (ELISA) (International Immuno-Diagnostics Inc, Foster City, CA, USA). Serum levels of vitamin D were also evaluated by ELISA (Roche Diagnostics, Indianapolis, IN, USA).

### 2.5. Statistical Analysis

Median and interquartile range are shown for continuous variables and counts and percentage for categorical variables. Categorical and continuous variables were compared by vitamin D group. *p*-values are unadjusted for multiple comparisons. 

## 3. Results

A total of 97 patients were enrolled in the study. Assessment of the patient’s clinical characteristics, overall, and by VDD, evaluated at the time of hospitalization, revealed the following (Table 1). Patient’s median (IQR) for age was 30.0 (22.0, 42.0) years and include 55 (56.7%) males. Serologically, most patients were either NS1+ or IgM+. All patients were within five days of dengue infection symptoms. The most prevalent symptoms were fever, body aches, vomiting, arthralgia, epistaxis, while less common symptoms include hematemesis, gum bleed, melena, hemoptysis, loose stools, hematuria, nausea, rectal and Per Vaginal (PV) bleed. In terms of disease severity, 37 (38.1%) patients were DF, 18 (18.5%) were DHF grade 1, 34 (35.0%) were DHF grade 2, seven (7.2%) were DHF grade 3 and one (1.0%) was DHF grade 4. Overall, 2 (2.1%) patients had a previous dengue infection (self-reporting), and 6 (6.2%) patients had a history of previous intake of vitamin D supplementation. 

**Figure 1:**
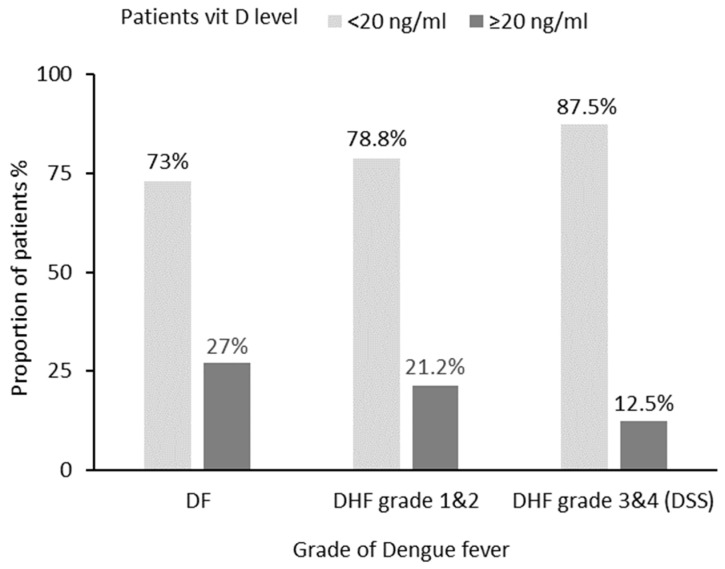
Prevalence of VDD by dengue stage on admission to hospital. Fisher’s exact *p*-value = 0.705.

Overall, the patient’s serum vitamin D level ranged from 4.2 to 109.7 ng/mL, and the median (IQR) was in the VDD range, i.e., 12.2 (9.1, 17.8) ng/mL (Table 1 and Table 2). Serum vitamin D median IQR in all the three dengue severity groups (DF, DHF, DSS) was in VDD range. The degree of VDD was relatively higher in the DSS patients as compared to DF and DHF grade 1 and 2 patients. In terms of VDD prevalence, majority of the patients in all the three dengue severity groups, i.e., 75 out of 97 (77.3%), were vitamin-D-deficient at the time of hospitalization, i.e., DF: 27 (73.0%), DHF grade 1 and 2: 41 (78.8%), and DHF grade 3 and 4 (DSS): 7 (87.5%) (Figure 1). 

In laboratory biochemistry (Table 3), evaluated at the time of hospitalization, 49 (86.0%) patients had markedly elevated serum levels of aspartate aminotransferase (AST), and alanine transaminase (ALT) liver enzymes, and the overall patients’ platelets count median (IQR) was also significantly low. In radiological examinations (Table 4), overall, 42 (43.2%) patients showed pericholecystic fluid and/or ascites in ultrasound, with more patients affected in VDD group. In circulatory system, 41 (43.2%) patients had a postural drop of ≥10 (mm Hg), with more patients in the vitamin D deficiency group. Patients’ hospitalization period was just under a week, i.e., median (IQR) 6.0 (3.0, 8.0) days, and in outcome, 96 (99%) were safely discharged from the hospital, and 1 (1.0%) patient (DHF grade 2, serum vitamin D level 11.02 ng/mL) was referred to nephrology for dialysis. 

## 4. Discussion

In this cohort of 97 patients hospitalized for dengue fever, most patients, i.e., 75 (77.3%) were vitamin-D-deficient at the time of hospitalization. We speculate that one potential cause of DF progression to severe illness in these patients could be their serum VDD which could not provide them adequate immune protection against the development of disease severe condition. Although due to small sample size, we could find a statistically significant difference in the dengue clinical manifestations between vitamin-D-deficient patients and those with vitamin D levels above the deficiency threshold (i.e., ≥20 ng/mL), some of the severe symptoms such as pericholecystic fluid/ascites were more prevalent in patients with VDD. It is possible that the severity of these symptoms may be linked with VDD. In this cohort analysis, consistent with the previously reported studies [18,19,20,21,22,23], 49 (86.0%) patients presented markedly elevated serum levels of both AST and ALT liver enzymes at admission. Acute phase of DENV infection is also associated with varying degrees of liver involvement, believed to result from hepatocyte apoptosis directly by the virus, hypoxic damage due to impaired liver perfusion resulting from fluid leakage, oxidative stress, or immune mediated injury [24,25,26]. Study by Hass et al. [27] has shown that vitamin D can inhibit endoplasmic oxidative stress, a pathophysiological condition reportedly activated during DENV infection, and is believed to be essential for DENV replication [28,29]. The authors had shown that vitamin D could down regulate the endoplasmic stress induced up-regulation of glucose-regulated protein 78 (GRP78), a protein that is essential for DENV replication. To date, no specific study has evaluated a possible link between VDD and markedly elevated liver enzymes levels in patients with DENV infection. In the present study, patients also had significantly low platelets count at admission. A rapid decline in platelet count is one of the indicators of clinical worsening and one of the warning signs of plasma leakage in dengue patients. The study also showed a postural drop of ≥10 mm Hg in 41 (42.2%) patients, which indicates compensated shock (pre-shock) which if not timely indicated and addressed can lead to frank shock with poor outcome. 

The possible role of vitamin D in DENV infection has been suggested by several studies [12,13,14,15,17,30], both in terms of its deficiency as a potential risk factor for developing severe dengue illness [31,32,33] and as possible adjuvant in the treatment of dengue patients [12,13,15,34,35]. Acute high dose vitamin D is believed to help in DF symptoms improvement and reduce the likelihood of progression to DHF/DSS. Some studies have shown contrasting results, i.e., higher serum 25-(OH) D is associated with more severe dengue (DHF/DSS) condition [11,16]. In the presence of limited studies, the available evidence on the possible association between VDD and susceptibility towards severe dengue illness is scanty and inconsistent. Though, VDD has been shown to induce increased susceptibility to viral infections including hepatitis C virus, influenza virus and human immunodeficiency virus (HIV) [36,37,38]. Few studies have shown the association of the vitamin D receptor (VDR) gene polymorphisms with susceptibility towards dengue virus infection [14,39]. 

The possible association of VDD and susceptibility towards severe DENV infection could be explained in several ways [40]. Vitamin D is a powerful immunomodulator affecting both the innate and adaptive immune responses [41] and plays a pivotal role in the pathogen-defense mechanisms [42]. Vitamin D activates both innate and adaptive immune response through several mechanisms including T-cells activation, macrophage differentiation and the production of anti-microbial peptides such as cathelicidin (LL-37) and β-Defensin [43,44,45]. Vitamin D also influences the expression of DENV entry receptor, dendritic cell specific intercellular adhesion molecule-grabbing non integrin (DC-SIGN) and FCγRIIA in immune cells [12,46,47]. Studies have also shown vitamin D antiviral activity against flaviviruses including hepatitis C virus [37,38,48,49] and DENV [11,12,13,15,50]. 

Our study is not free from limitations, which could be addressed in future studies. We did not have a control group of non-dengue febrile patients or healthy volunteers. Additionally, our study involved small sample size, as this was an exploratory study, hence, our study was not sufficiently powered to examine the effects of 25-(OH) D on different subgroups of patients and severity indicators of dengue illness. Nevertheless, we have carried out a pragmatic study which suggest that VDD may be a possible risk factor towards susceptibility of severe DENV illness. The results of this study are generalized, widely applicable and can help raise public awareness in people living in endemic areas against severe DENV infection.

## 5. Conclusions

Our study suggests a possible association between low systemic 25-(OH) D levels and increased risk of severe dengue illness in people living in the endemic areas. Regular testing of serum vitamin D levels is highly encouraged, and in case of VDD, medical advice should be sought, and followed. Overcoming VDD can possibly help in protection against severe DENV illness. 

## Figures and Tables

**Table 1:** Patients’ demographic and clinical characteristics, overall and by vitamin D levels, at the time of hospitalisation.

Clinical Symptom	Overall (n = 97)	Patients with Vit D <20 ng/mL (n = 75)	Patients with Vit D ≥20 ng/mL (n = 22)	*p*-Value
Age, median (IQR), years	30.0 (22.0, 42.0)	30.0 (21.5, 39.0)	35.0 (30.0, 50.0)	0.026
Gender, male, n%	55 (56.7)	43 (57.3)	12 (54.5)	0.812
Serology (n%)				
NS1+	28 (28.9)	21 (28.0)	7 (31.8)	0.617
NS1+, IgM+	8 (8.2)	8 (10.7)	0 (0.0)	
IgM+	44 (45.4)	33 (44.0)	11 (50.0)	
IgM+, IgG+	16 (16.5)	12 (16.0)	4 (18.2)	
NS1+, IgM+, IgG+	1 (1)	1 (1.3)	0 (0.0)	
Dengue presentations, median (IQR) days	5.0 (5.0, 6.0)	5.0 (5.0, 6.0)	5.0 (5.0, 5.0)	0.293
Dengue Symptoms (n%)				
Fever	97 (100.0)	75 (100.0)	22 (100.0)	>0.99
Bodyaches	84 (86.6)	64 (85.3)	20 (90.9)	0.726
Abdominal pain	18 (18.6)	14 (18.7)	4 (18.2)	>0.99
Headache	20 (20.6)	16 (21.3)	4 (18.2)	>0.99
Epistaxis	15 (15.5)	13 (17.3)	2 (9.1)	0.508
Vomiting	14 (14.4)	11 (14.7)	3 (13.6)	>0.99
Arthralgia	10 (10.3)	5 (6.7)	5 (22.7)	0.044
Hematemesis	5 (5.2)	3 (4.0)	2 (9.1)	0.317
Gum bleed	4 (4.1)	3 (4.0)	1 (4.5)	>0.99
Malena	3 (3.1)	3 (4.0)	0 (0.0)	>0.99
Haemoptysis	3 (3.1)	3 (4.0)	0 (0.0)	>0.99
Loose stools	3 (3.1)	2 (2.7)	1 (4.5)	0.542
Hematuria	1 (1.0)	1 (1.3)	0 (0.0)	>0.99
Nausea	2 (2.1)	1 (1.3)	1 (4.5)	0.403
Rectal bleeding	2 (2.1)	2 (2.7)	0 (0.0)	>0.99
PV Bleeding	2 (2.1)	2 (2.7)	0 (0.0)	>0.99
Others	3 (3.1)	3 (4.0)	0 (0.0)	>0.99
Previous Dengue infection, n%	2 (2.1)	2 (2.7)	0 (0.0)	>0.99
Previous vitamin D intake, n%	6 (6.2)	2 (2.7)	4 (18.2)	0.022
Vitamin D level, median (IQR), ng/mL	12.2 (9.1, 17.8)	10.6 (8.4, 12.7)	28.9 (25.7, 42.5)	

n: numbers of patients. n (%) and median (IQR) are shown for categorical and continuous measures, respectively.

**Table 2:** Serum vitamin D levels at the time of hospitalization.

Dengue Fever Severity	Serum Vitamin D Level, Median (IQR) ng/mL
Overall (n = 97)	12.2 (9.1, 17.8)
DF (n = 37)	12.5 (8.1, 20.2)
DHF grade 1 and 2 (n = 52)	12.4 (10.3, 17.7)
DHF grade 3 and 4 (DSS) (n = 8)	9.5 (7.9, 11.9)

n: numbers of patients.

**Table 3:** Patient’s laboratory biochemistry.

Biochemistry	Overall (n = 97)	Patients with Vit D <20 ng/mL (n = 75)	Patients with Vit D ≥20 ng/mL (n = 22)	*p*-Value
Liver enzymes	n = 57	n = 47	n = 10	
Abnormal Bilirubin, n%	13 (22.8)	11 (23.4)	2 (20.0)	>0.99
Median (IQR), mg/dL	0.6 (0.4, 0.9)	0.7 (0.4, 0.9)	0.6 (0.5, 1.1)	0.723
Abnormal AST, n%	49 (86.0)	40 (85.1)	9 (90.0)	>0.99
Median (IQR), IU/L	132.0 (73.0, 194.0)	131.0 (73.0, 186.5)	143.0 (124.8, 385.2)	0.341
Abnormal ALT, n%	49 (86.0)	40 (85.1)	9 (90.0)	>0.99
Median (IQR), IU/L	73.0 (47.0, 139.0)	70.0 (46.0, 132.5)	80.0 (54.5, 211.5)	0.393
Complete blood count				
Haemoglobin, median (IQR), g/dL	13.2 (12.0, 14.5)	13.1 (12.0, 14.5)	13.7 (12.3, 14.7)	0.725
Total leukocyte count, median (IQR) × 10^9^/L	4.7 (3.8, 6.2)	4.6 (3.8, 6.0)	5.8 (4.0, 7.4)	0.470
Haematocrit, median (IQR) %	43.0 (41.0, 47.0)	43.6 (41.0, 47.5)	43.0 (41.0, 46.2)	0.285
Platelets, median (IQR) × 10^9^/L	30.5 (20.0, 53.0)	30.5 (20.0, 51.5)	30.5 (21.0, 55.0)	0.681
Neutrophil, median (IQR) %	54.1 (43.0, 68.0)	54.0 (43.5, 68.0)	58.8 (43.2, 67.0)	0.963
Lymphocyte, median (IQR) %	31.5 (24.0, 39.0)	31.6 (24.0, 39.0)	30.4 (24.2, 41.5)	0.724

n: numbers of patients. Reference range: Bilirubin: 0.2–1.2 mg/dL; AST: 1–45 IU/L; ALT: 0–40 IU/L, Haemoglobin: 13.6–16.2; Leukocyte count: 4.5–11 × 10^9^; haematocrit: 41–50%; platelets count: 150–450 × 10^9^/L; Neutrophile: 50–70%; Lymphocyte: 20–40%.

**Table 4:** Radiology and circulatory disease parameters by vitamin D deficiency status.

Clinical Characteristic	Overall (n = 97)	Vitamin D <20 ng/mL (n = 75)	Vitamin D ≥20 ng/mL (n = 22)	*p*-Value
Chest x-ray (n%)				
Right-side fleural effusion	6 (6.2)	4 (5.3)	2 (9.1)	-
Bilateral fleural effusion	2 (2.1)	1 (1.3)	1 (4.5)	-
Unremarkable	89 (91.8)	70 (93.3)	19 (86.4)	0.346
Ultrasound (n%)				
Pericholecystic fluid	16 (16.5)	12 (16.0)	4 (18.2)	-
Ascites	9 (9.3)	8 (10.7)	1 (4.5)	-
Pericholecystic fluid + Ascites	26 (26.8)	23 (30.7)	3 (13.6)	-
Hepatomegaly/hepatosplenomegaly	2 (2.1)	2 (2.7)	0 (0.0)	-
Unremarkable	43 (44.3)	29 (38.7)	14 (63.6)	0.357
Circulatory system				
Pulse rate, BPM, median (IQR)	90.0 (90.0, 100.0)	92.0 (90.0, 100.0)	90.0 (81.8, 100.0)	0.309
Systolic BP, median (IQR) (mm Hg)	110.0 (100.0, 120.0)	110.0 (100.0, 117.5)	110.0 (101.2, 120.0)	0.728
Diastolic BP, median (IQR) (mm Hg)	70.0 (60.0, 80.0)	70.0 (60.0, 80.0)	70.0 (61.2, 80.0)	0.765
Postural drop ≥ 10 (mm Hg)	41 (42.2)	36 (48.6)	5 (23.8)	0.049
CRT ≥ 2 s n%	10 (10.3)	9 (12.0)	1 (4.5)	0.447

n: numbers of patients.

## Data Availability

The data that support the findings of this study are available from the corresponding author upon reasonable request.
